# Synthesis and Characterization of Ti–Nb Alloy Films Obtained by Magnetron Sputtering and Low-Energy High-Current Electron Beam Treatment

**DOI:** 10.3390/ma14123238

**Published:** 2021-06-11

**Authors:** Federico Morini, Massimiliano Bestetti, Silvia Franz, Antonello Vicenzo, Alexey Markov, Evgeniy Yakovlev

**Affiliations:** 1 Department of Chemistry, Materials and Chemical Engineering “G. Natta”, Politecnico di Milano, 7 Via Luigi Mancinelli, 20131 Milano, Italy; massimiliano.bestetti@polimi.it (M.B.); silvia.franz@polimi.it (S.F.); antonello.vicenzo@polimi.it (A.V.); 2 The Weinberg Research Center, Tomsk Polytechnic University, 30 Lenin Ave, 634050 Tomsk, Russia; 3 Tomsk Scientific Center SB RAS, 10/4 Akademicheskii Pr, 634055 Tomsk, Russia; almar@lve.hcei.tsc.ru (A.M.); yakov_e@mail.ru (E.Y.)

**Keywords:** titanium–niobium alloys, graded alloy, low-energy high-current electron beam

## Abstract

The aim of the present work is to investigate the synthesis of Ti–Nb alloy films obtained by the physical vapor deposition (PVD) magnetron sputtering of Nb films on Ti substrates, followed by low-energy high-current electron beam (LEHCEB) alloying treatment. Ti–Nb alloys were synthetized under two different regimes, one by varying the deposited amount of Nb (from 25 to 150 nm) and treating samples with low applied voltages and a number of pulses (three pulses at either 20 or 25 kV), the second by setting the amount of Nb (100 nm) and alloying it at a higher applied voltage with a different number of pulses (from 10 to 50 at 25 and 30 kV). The synthetized Ti–Nb alloys were characterized by XRD and GDOES for phase identification and chemical composition; SEM and optical microscopy were employed for morphology evaluation; compositional investigation was done by EDS analysis and mechanical properties were evaluated by microindentation tests. LEHCEB treatment led to the formation of metastable phases (α′, α″ and β) which, together with the grain refinement effect, was responsible for improved mechanical properties.

## 1. Introduction

Ti alloys are attractive materials for their low density, excellent corrosion resistance and good mechanical properties, even though they are characterized by low wear resistance [1]. In the last decade, attention has been focused on improving the surface properties of Ti and its alloys for biomedical applications. The best results were achieved by alloying Ti with *d*-metals such as V, Ta, Mo and Nb [2,3]. Compared to Ti6Al4V, Ti–Nb alloys have better wear resistance and fewer health-related issues [4,5]. By controlling the Nb content and the melt-cooling rate it is possible to obtain metastable phases α′, α″, ω and β phase to improve the mechanical properties. Such phases are formed by the rapid cooling of the β phase. The α′ phase is obtainable by adding a low amount of Nb, and it is characterized by fine acicular microstructure. By increasing the content of the alloying element, an orthorhombic α″ phase forms, with a finer acicular microstructure and higher mechanical properties [1]. The hexagonal ω phase can be formed only when the content of Nb is between 26 and 34 at.%. Above this range, the β phase is formed [6,7].

As reported by Lee et al. [1], Ti–Nb alloys have a higher Vickers hardness (HV) than commercially pure (c.p.) Ti. In particular, alloys containing the α′ phase have higher microhardness, usually around 340 HV, while those containing the α″ phase have a hardness of around 285 HV. On the other hand, the β-phase alloys with a higher Nb content have a lower hardness. The presence of the ω phase leads to the highest hardness obtainable, around 375 HV. Similar results were also observed in other binary systems such as Ti–Mo, Ti–Ta, Ti–V and Ti–Zr [8,9,10].

**Figure 1:**
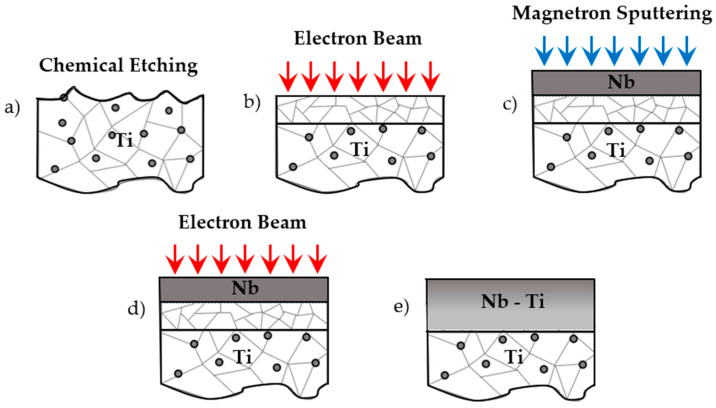
Scheme of the treatment cycle (**a**–**e**).

Several techniques can be used to synthetize Ti–Nb, such as the electrodeposition, melt casting, ion mixing or mechanical alloying of elemental powders [11,12,13,14,15]. The low-energy high-current electron beam (LEHCEB) technique has been investigated for several years for surface modification and, more recently, for producing surface alloys [16]. It allows the modification of the composition and microstructure of the surface layer by rapid melting and solidification (10^9^ K/s) [17]. This leads to grain refinement and element recombination, with the formation of metastable phases [18]. Mechanical and corrosion resistance properties can also be enhanced [19]. LEHCEB coupled with PVD magnetron sputtering allows for the synthesis of surface alloys with a graded and non-equilibrium composition [20]. The final thickness of the resulting alloy depends on the amount of deposited material during the sputtering process, and on the energy delivered with the pulsed electron beam (EB). In the present work, compositionally graded Ti–Nb alloys were prepared according to the process scheme in Figure 1, by magnetron sputtering deposition of Nb thin film followed by LEHCEB treatment. The interdiffusion of Nb into the bulk Ti was investigated together with mechanical and crystallographic properties.

## 2. Materials and Methods

### 2.1. Material Preparation

Ti grade I (c.p.) was used as the material for substrates, containing O ≤ 0.08%, C ≤ 0.08 and Fe < 0.07. Titanium samples (3 × 6 × 0.2 cm^3^) were degreased with ultrasonic treatment in acetone and ethanol solution and chemically etched for 2 min in an aqueous solution of 2 M HF and 1.44 M HNO_3_ at room temperature, rinsed in deionized water and nitrogen dried. After chemical preparation, all substrates underwent an electron beam (EB) pretreatment. The utilized RITM-SP facility (“Microsplav OOO”, Tomsk, Russia) is equipped with three direct-current (DC) magnetron sputtering sources and an explosive emission cathode able to emit an EB with low energy and high current. Selected operating parameters are summarized in Table 1.

A first set of Ti–Nb surface alloys (set I) was synthetized by depositing 25, 50, 75, 100, 125 and 150 nm of Nb on Ti substrates. Magnetron sputtering deposition was carried out with a Nb (99.9999%) target of 75 mm in diameter, with a constant applied current (1 A and 315 V). It was followed by EB alloying in two conditions: three pulses of either 20 kV or 25 kV. Table 2 summarizes the alloying procedure for each sample.

A second set of samples (set II) was synthetized, depositing 100 nm of Nb and using between 10 and 50 EB pulses at 25 or 30 kV (Table 3).

### 2.2. Sample Characterization

Optical microscopy was carried out with a Leica DMLM optical microscope equipped with a DFC290 camera (Leica Camera, Wetzlar, Germany). The surface roughness parameters (average roughness Ra and root mean square roughness Rq) were measured with a UBM Microfocus laser profilometer (Messtechnik GmbH, Ettlingen, Germany) over a 1.25 mm path (acquisition rate 1400 pts/mm). Surface morphology was assessed with scanning electron microscopy (SEM, ZEISS EVO 50VP, Carl Zeiss Jena GmbH, Jena, Germany). Energy-dispersive X-ray spectroscopy (EDS) measurements, performed with a Bruker Quantax 200 6/30 spectrometer (Bruker Corp., Billerica, MA, USA), provided an averaged surface alloy composition of samples. The crystalline structure assessment was done by X-ray diffraction (XRD) analysis with an EMPYREAN PW1830 diffractometer (PANalytical Ltd., Malvern, UK and Amelo, The Netherlands), in Bragg–Brentano and grazing incidence angle (1°) configurations (Cu Kα1, λ = 0.15406 nm). Peak fitting and deconvolution were performed using the fitting function Pearson VII provided by OriginPro2018 (Origin Lab Corporation, Northampton, MA, USA). Glow-discharge optical emission spectroscopy (GDOES) was performed using a GDA750 Analyzer (Spectruma Analytik GmbH, Hof, Germany), to measure the elemental depth profile composition. Microhardness measurements were made with a FischerScope HVX100 (load 10–500 mN) (Helmut Fischer GmbH, Sindelfingen, Germany) to determine Vickers microhardness, maximum penetration depth and elastic modulus.

## 3. Results and Discussion

### 3.1. Morphological Analysis

#### 3.1.1. Optical Microscopy

**Figure 2:**
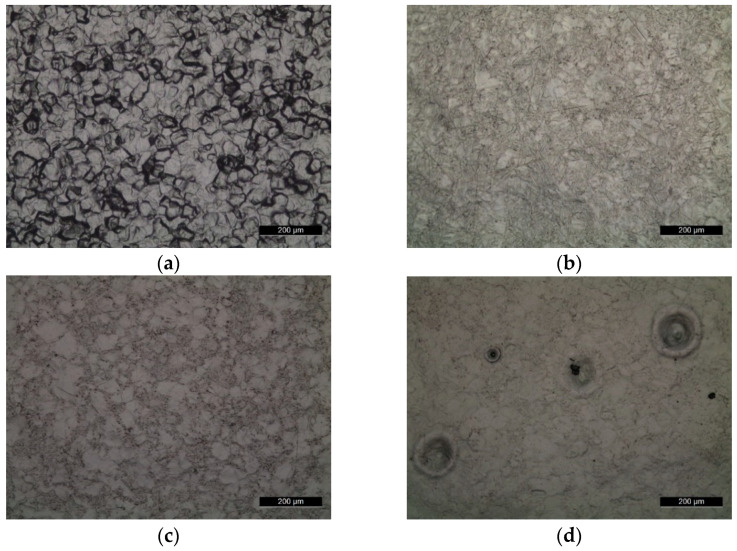

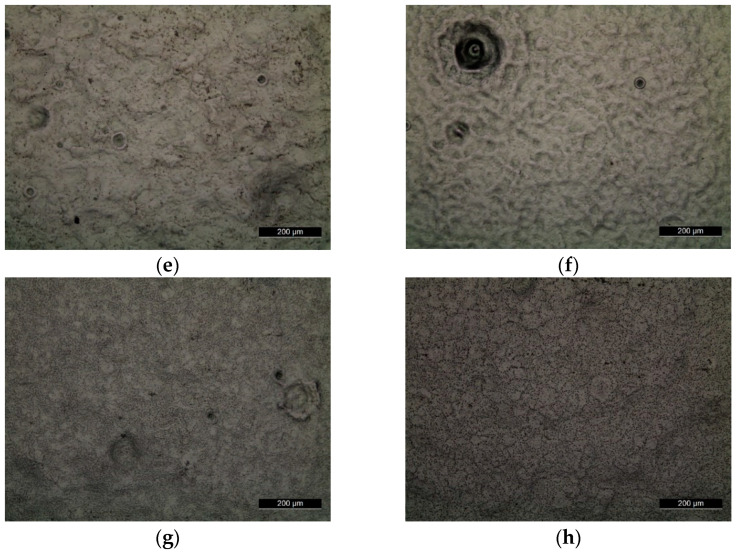
Optical micrographs of samples: (**a**) Ti-etched, (**b**) Ti-EB, (**c**) 1, (**d**) 2, (**e**) 5, (**f**) 6, (**g**) 11 and (**h**) 12.

Optical surface micrographs showed that the chemical pretreatment of the Ti substrate induced grain-boundary etching (Figure 2a). However, after EB pretreatment (Figure 2b) the surface appeared smoother and grain refinement could be inferred. Depending on the amount of deposited Nb, the number of EB pulses and the accelerating voltage, different morphologies were obtained. As shown in Figure 2c,e,g, by increasing the amount of Nb deposited on the Ti substrate (25, 75 and 150 nm), better coverage and homogeneity of the alloyed layers were observed. Correspondingly, by increasing the acceleration voltage from 20 to 25 kV, more craters and shrinkage voids formed (Figure 2d,f,h). Pits and craters were present due to the ejection of impurities within the substrate. One way to avoid such defects is to increase the number of pulses, as reported by Rotshtein et al. [16] and by Ozur et al. [21]. The size and density of the craters depend on the sample purity and the delivered energy. A higher number of craters leads to an increase of roughness and non-uniform microstructure, with consequent reduction of the mechanical properties of the material [22]. Roughness values (Table 4) and optical micrographs (Figure 2) confirm the smoothing effect due to the LEHCEB treatment.

After LEHCEB it was possible to have a more homogenous surface with a finer microstructure (Figure 2b). In the sample treated at a higher applied voltage there were more craters (Figure 2d–f). Differences in morphology were also due to the amount of the deposited Nb; at a higher applied energy the Nb was completely melted. On the other hand, it is more difficult to reach the melting and the homogenization of Nb with Ti by applying lower energy.

#### 3.1.2. SEM and EDS

**Figure 3:**
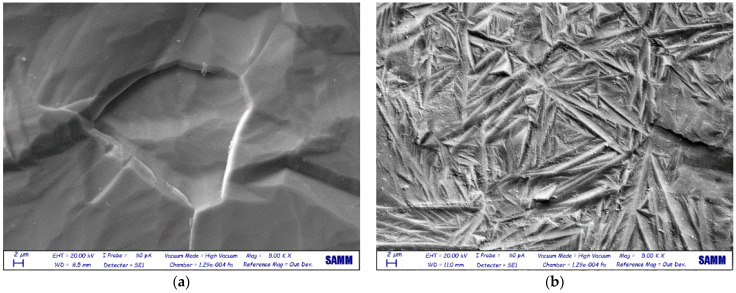
SEM images of (**a**) etched and (**b**) EB-treated Ti samples.

The surface of Ti after EB treatment was characterized by a fine acicular microstructure, compared with the untreated samples (Figure 3). This is due to the LEHCEB treatment, which leads to a rapid solidification process [10]. The surface morphology of Figure 3b corresponds to a martensitic transformation, as described in the literature [19].

**Figure 4:**
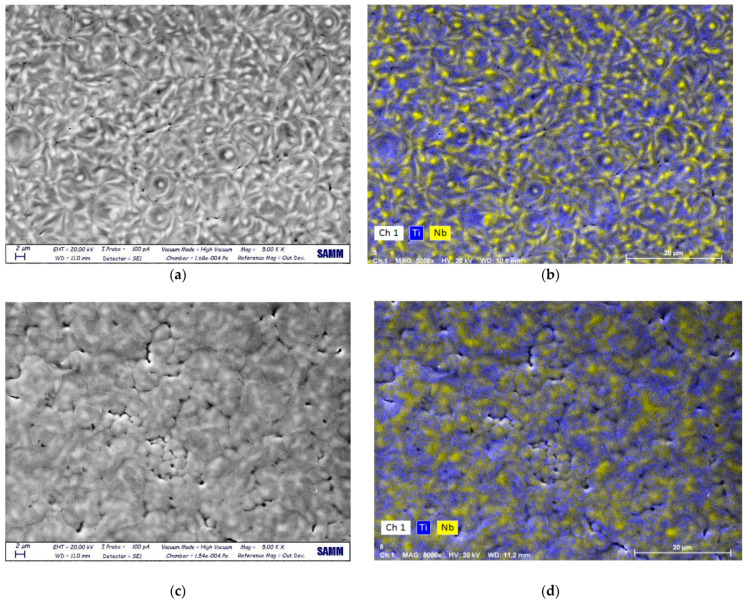
SEM images and EDS maps (Ti, Nb) of sample 5 (**a**,**b**) and sample 6 (**c**,**d**), respectively.

Alloy thin films made with the same amount of Nb had different microstructures and morphologies according to the delivered energy. In sample 5 (three pulses at 20 kV) (Figure 4a) Nb did not undergo a complete melting and mixing with the substrate. EDS maps of Nb and Ti in Figure 4b show the inhomogeneous distribution of Nb.

**Figure 5:**
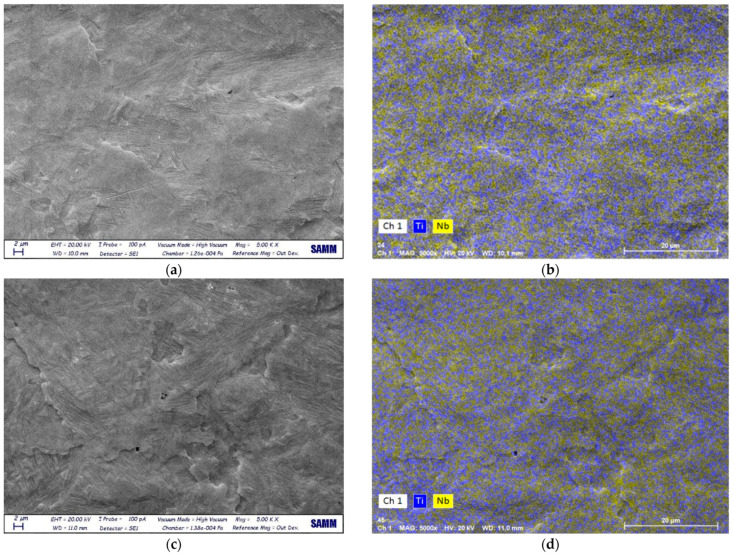
SEM images and EDS maps (Ti, Nb) of sample 16 (**a**,**b**) and sample 20 (**c**,**d**), respectively.

On the other hand, more homogeneity was found in sample 6 (Figure 4c), characterized by a granular morphology. The size of the acicular grains was smaller than the ones observed in the Ti-EB sample due to the higher energy provided with EB treatment. There were microcracks and shrinkage voids due to the rapid solidification process. Concerning EDS results, the Nb content was 8.2 at.% in sample 5 and 6.5 at.% in sample 6. These higher mean values were due to the presence of islands of unmixed Nb. This difference could be attributed to the alloying LEHCEB process; a less-intense treatment (i.e., at 20 kV) led to the incomplete melting of the deposited Nb layer, without the possibility of mixing with the underlying Ti. By increasing the applied voltage, a more homogeneous and deeper interdiffusion of the two elements was obtained. Samples of set II had different morphologies compared to the samples of set I, and were characterized by a finer structure (Figure 5). In sample 16, Nb was melted and mixed with the substrate. The effect of LEHCEB was then to smooth and reduce the grains, as seen in Figure 5a. In sample 20 there was the same fine structure, with smaller and more defined grains (Figure 5c). This effect was due to the presence of Nb, which led to the formation of the α′ phase. A high applied voltage (30 kV) led to complete melting of Nb, inter-diffusion with the substrate, reduction of crystallite size and formation of a finer microstructure. Considering the chemical composition as assessed by EDS, samples treated at a higher voltage and higher number of pulses showed a lower content of Nb on the surface, even if the deposited amount was higher (100 nm). This was 5.5 and 4.9 at.% for a sample synthetized at 30 kV with 20 or 50 pulses, respectively. This was correlated to the high applied voltage, since in samples treated at 20 kV and 25 kV there was an incomplete melting and alloying of Nb with Ti, and then a higher content of Nb in the surface layer, while at 30 kV it was possible to mix the two elements, and obtain a larger diffusion depth into the substrate.

### 3.2. Surface Characterization

#### 3.2.1. XRD

**Figure 6:**
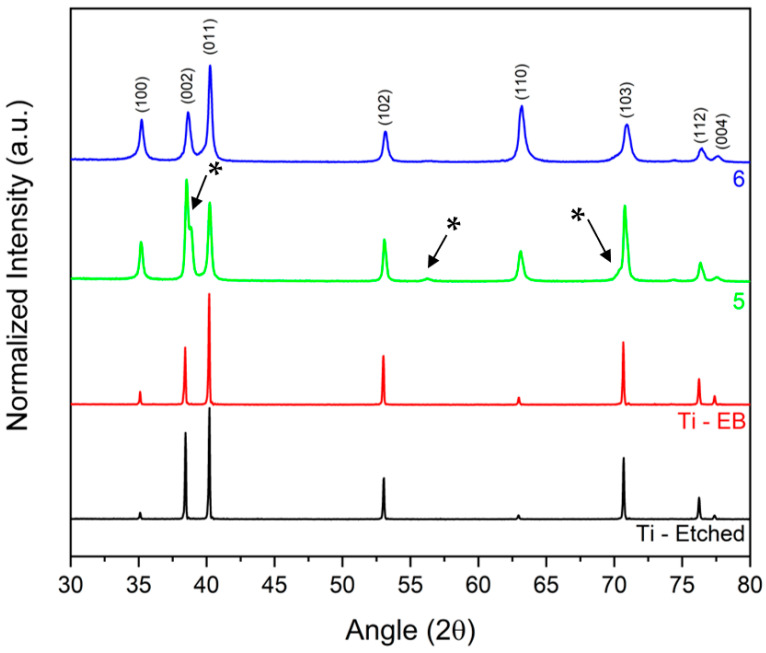
XRD pattern of etched and EB-treated Ti and samples 5 and 6 (Bragg–Brentano). The peaks of a distorted α″ orthorhombic phase are indicated by the asterisk (*) symbol.

The XRD patterns of etched and EB-treated Ti samples are shown in Figure 6. The calculated lattice parameters for etched Ti are *a* = 2.949 Å and *c* = 4.682 Å (*c*/*a* = 1.588), while for EB-treated Ti they are *a* = 2.949 Å and *c* = 4.679 Å (*c*/*a* = 1.587). The Ti-EB sample shows slight changes in peak intensity, which stands for a surface crystallographic texture modification [19]. The Scherrer equation (L = Kλ/Bcosθ, where *L* is the crystallite size, *K* = 0.94 is the shape factor, *λ* the X-ray wavelength, *B* the FWHM and *θ* the Bragg’s angle) allows the estimation of crystallite size (Table 5). Expectedly, as a result of alloying with Nb, the characteristic peaks of the α′ phase of samples 5 and 6 are less intense, broader and shifted with respect to Ti-EB the Nb content in α′ *hcp* structure, calculated from lattice constant vs. composition data [23], was around 5.5 at.%.

The XRD pattern of sample 5 shows the presence of peaks (38.85°, 56.26° and 70.81°) that could be attributed to a distorted α″ orthorhombic phase, with a higher amount of Nb. The presence of a phase richer in Nb is also confirmed by the EDS maps, where Nb-rich regions are visible.

**Figure 7:**
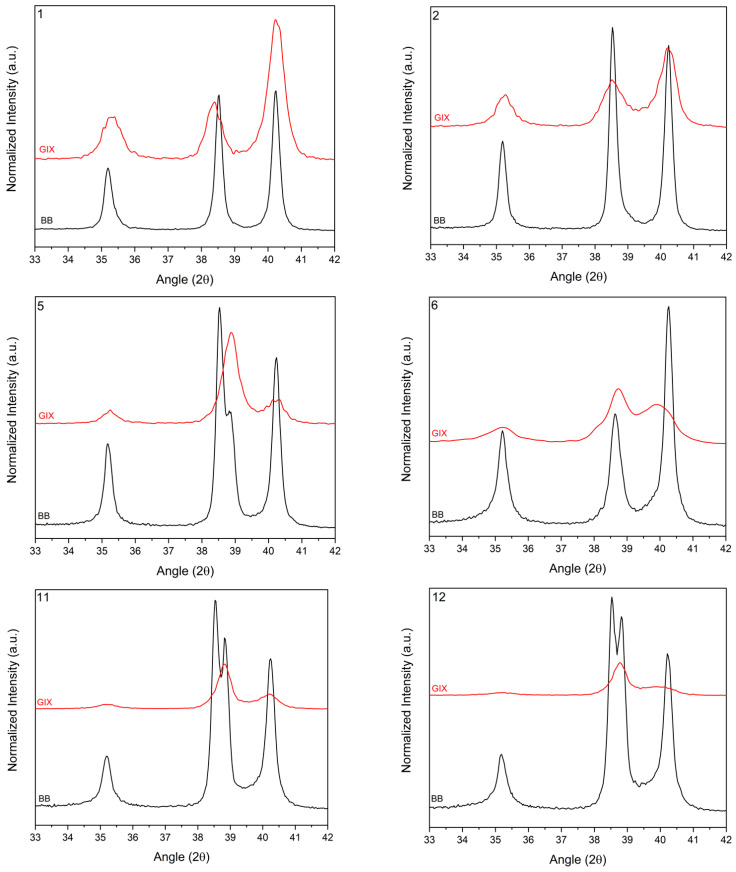
Grazing incidence angle (GIX) vs. Bragg–Brentano (BB) spectra of set I samples, as indicated by the number in the upper left corner of the plots.

**Figure 8:**
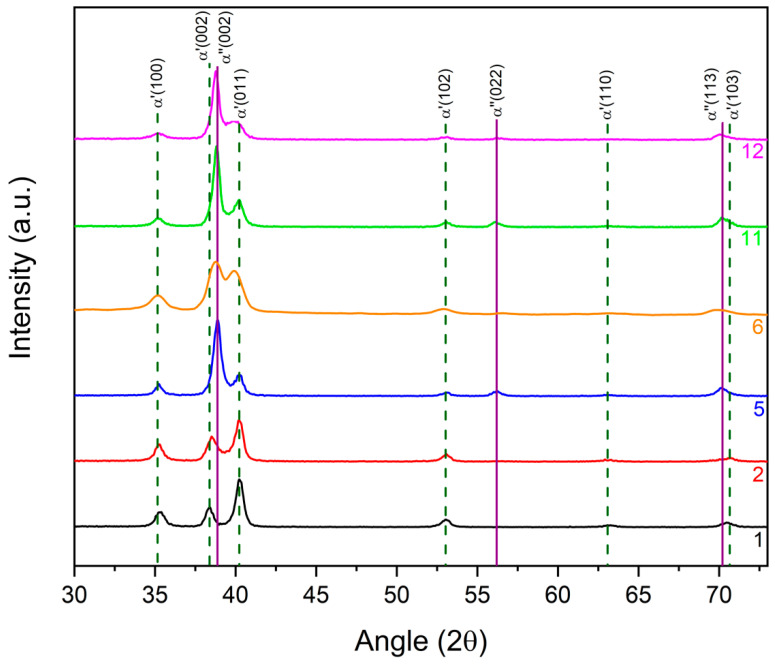
XRD patterns of samples 1, 2, 5, 6, 11 and 12 (grazing incidence angle).

In grazing incidence angle XRD spectra, peaks that could not be attributed to an α′ *hcp* structure were attributed to α″ orthorhombic structure (Figure 7). From peak positions, as derived from XRD patterns shown in Figure 8, orthorhombic lattice parameters were estimated, as reported in Table 6. The surface alloy of set I samples seemed to be characterized by an external layer of α″ and a deeper α′ phase, both with a composition independent from the initial deposited Nb thickness. This was likely due to the low EB energy and low number of pulses delivered to set I samples. The Nb content in this surface film was estimated to be between 22 and 24 at.%, considering the orthorhombic phase. Lattice parameters and volume (V=abc) are reported in Table 6 [23,24].

**Figure 9:**
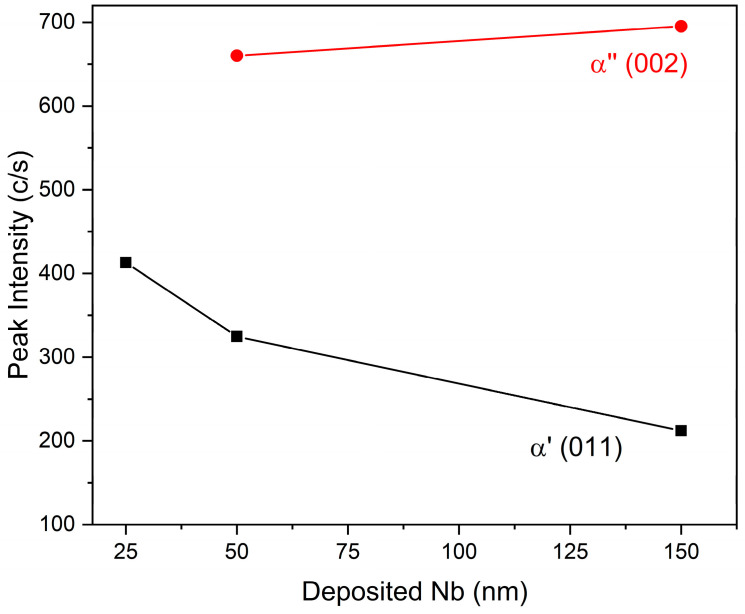
Peak intensity of α′ (011) and α″ (002) as a function of deposited Nb.

Samples of set I were likely differentiated by the thickness of the external layer. Indeed, from Figure 9, the intensity of *hcp* peaks was attenuated by increasing the thickness of the deposited Nb, while the intensity of the α″ phase peaks was increased.

**Figure 10:**
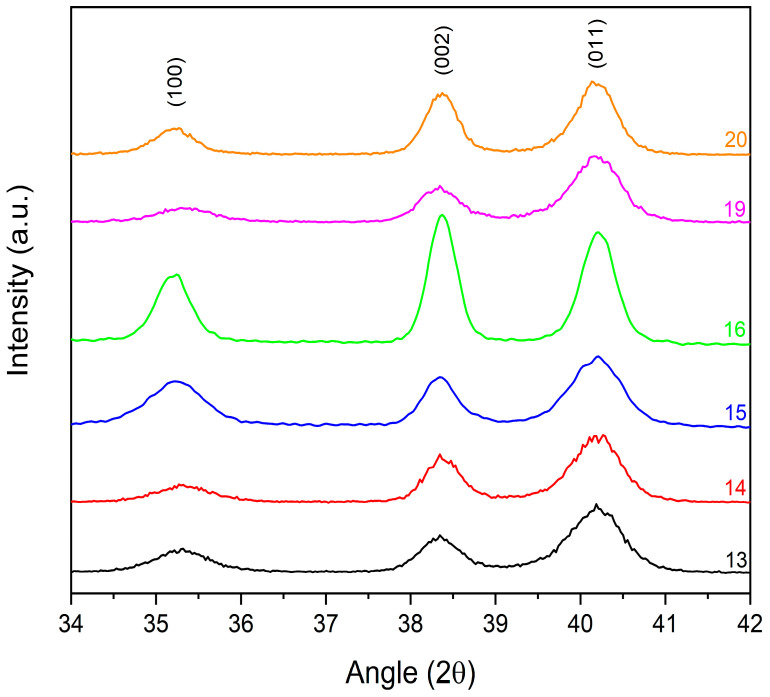
XRD patterns of samples of set II (grazing incidence angle).

Different results were obtained with samples of set II. A higher applied voltage and number of pulses promoted the melting and the interdiffusion of Nb and Ti, with the formation of an α′ *hcp* Ti–Nb alloy (Figure 10). Lattice parameters of the α′ *hcp* and cell volume ($V=a^{2}csen$60°) were calculated from the XRD reflections in a Bragg–Brentano configuration and are listed in Table 7 for all samples with *hcp* structure.

The Nb content in the alloy, calculated from peak positions, was around 6 at.% (Table 7), which is to be compared with the equilibrium value of *hcp* structure—that is, around 2 at.% [23,25]. Another effect of the high number of pulses at a higher applied voltage was the further decrease in the crystallite size, which diminished even more compared to the first set of samples. In samples of set II, crystallites reached a mean size of around 15 nm.

#### 3.2.2. GDOES

**Figure 11:**
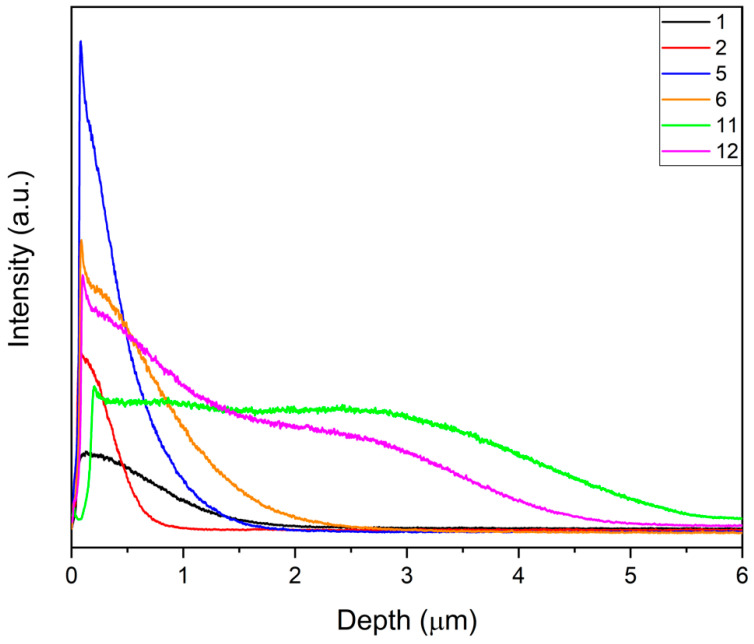
Comparison of Nb GDOES profiles of different samples.

A high Nb intensity signal was observed at the surface of the alloy with a graded composition. The Nb profile depended on the total amount of metal deposited, the applied voltage and the number of pulses. Minimum penetration of Nb in the substrate was only 0.4 μm in sample 2 (25 nm). On the other hand, it reached almost 4 μm in sample 11, when the Nb film was 150 nm (Figure 11). Brunella et al. [10] reported that a high number of pulses (set II) is the main reason for better homogenization, while a higher applied voltage leads to a thicker modified layer.

The mean Nb content (at.%) was estimated in a different surface by considering the initial deposited amount of Nb and the thickness of the final alloy, as determined by the inflection point in GDOES profiles (Table 8). Samples with a more intense GDOES signal at the surface were characterized by the presence of an outermost orthorhombic α″ phase.

**Figure 12:**
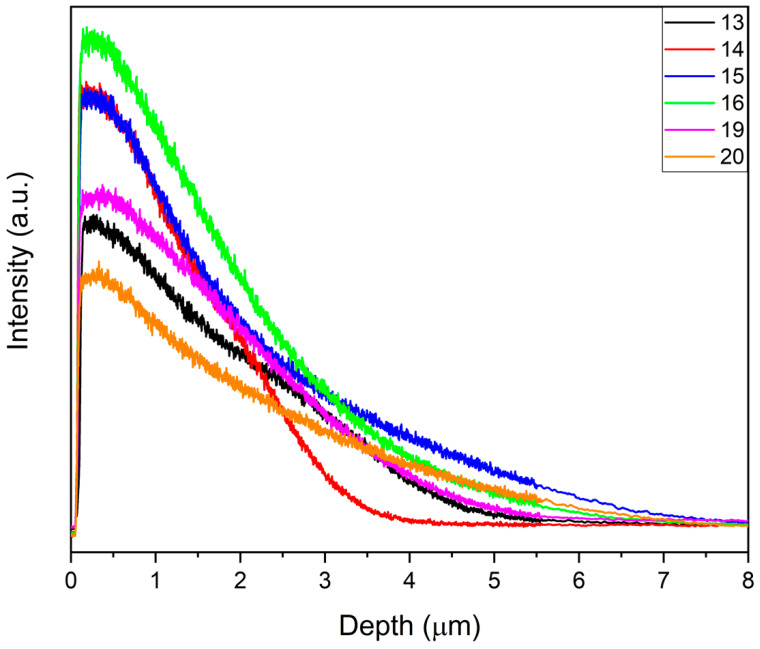
Comparison of Nb GDOES profiles of different samples of the second set.

EB treatments performed on 100 nm films of Nb (set II), at a higher energy and number of pulses, led to a deeper modified layer (Figure 12). Samples of set II were more homogeneous, with a more even Nb gradient composition (Table 9). The Nb mean content had a maximum value of about 6.8 at.% for sample 20. This was the sample with a relatively shorter diffusion depth of Nb, which was more concentrated in a thinner surface layer, as confirmed by XRD analysis.

### 3.3. Microhardness Tests

**Figure 13:**
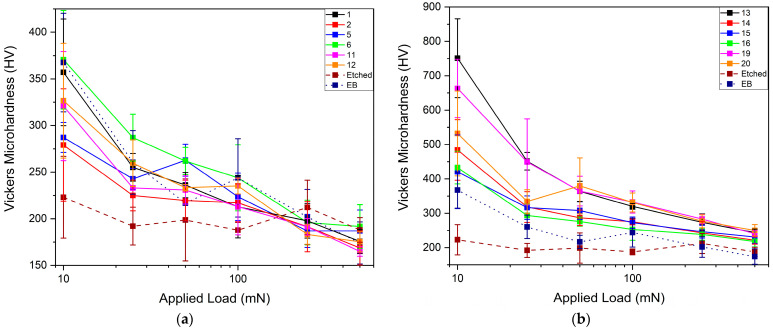
Vickers microhardness as a function of applied load for samples of the first (**a**) and second set (**b**), respectively.

Vickers microhardness tests were conducted on selected samples of set I and set II, together with etched and EB-treated Ti (Figure 13). Microhardness values at high loads converged to 170–190 HV in samples of set I, and to 230 HV in the case of set II. This was due to the presence of Nb, which interdiffused in a deeper layer in the samples of set II, influencing the mechanical properties of the Ti substrate [26]. Similar results were observed by Zhang et al. [19], who explained the formation of ultrafine grains, produced after fast solidification, which led to a higher surface hardness. The highest surface microhardness was obtained in set I for sample 6 (370 HV at 10 mN). Concerning the phase composition, the mechanical properties could increase with the decrease of the α phase content and the formation of finer metastable phases. Microhardness increased due to the presence of Nb alloyed with Ti. The samples with a higher content of the orthorhombic phase α″ had a lower microhardness than those with a higher content of harder martensitic phase α′ [27,28]. Reduction in crystallite size led to an increase in microhardness.

There was a further increase in microhardness by treating samples at a higher applied voltage and number of pulses (Figure 13b). In particular, all samples reached values from 450 to 750 HV at an applied load of 10 mN. Treatments at 30 kV led to a lower increase than those at 25 kV because of the cratering effect: there is the possibility of having a higher number of craters by increasing the energy, which could be decreased by increasing the number of pulses [29]. Another factor for higher hardness is the crystallite size: samples treated at 25 kV had a smaller size compared to others. The high increase in microhardness was connected to the formation of the Ti–Nb alloy, in which it is possible to have a higher content of Nb on the surface, and to the presence of α′ phase, also confirmed by XRD patterns.

## 4. Conclusions

Ti–Nb surface alloys were produced using PVD and LEHCEB techniques in different experimental conditions. Nb layers with thickness between 25 and 150 nm were deposited onto c.p. Ti grade I, and different accelerating voltages (20, 25 and 30 kV) were applied to produce a surface alloy. LEHCEB treatments performed at a higher applied voltage and number of pulses allowed for a better homogenization of the surface layer, with smoother and finer morphology. XRD results showed the presence of different metastable phases, namely, α′ *hcp* martensitic and α″ orthorhombic phases. Samples of set I were characterized by an external orthorhombic layer (from 22 to 24 at.% of Nb) and an internal *hcp* α′ alloy (from 5.40 to 5.55 at.%), while the samples of set II showed the presence of only an α′ *hcp* phase (from 5.60 to 6.35 at.%). Elemental EDS maps showed that samples produced at a lower voltage (20 kV) were less homogeneous than those treated at a higher voltage (30 kV), with an Nb mean content around 5.5 at.%. Using GDOES, it was possible to assess the thickness of surface alloys, which reached around 1.5 μm, and the mean Nb content was evaluated to be around 6 at.%, forming a compositionally graded alloy on the surface. Metastable phases, together with crystallite size reduction, led to an increase of surface microhardness, reaching values of 370 HV (set I) and 750 HV (set II) at a low applied load (10 mN).

## Figures and Tables

**Table 1:** EB operating conditions for pretreatment of the samples.

Parameters	Values
Energy of electrons (keV)	27
Pulse duration (μs)	2–4
Number of pulses	10
Beam energy density (J/cm^2^)	3.5
Pulse frequency (Hz)	0.2
Ar pressure (Pa)	0.02

**Table 2:** Alloying parameters for samples of set I.

Sample	1	2	3	4	5	6	7	8	9	10	11	12
PVD (Nb, nm)	25	25	50	50	75	75	100	100	125	125	150	150
EB (kV)	20	25	20	25	20	25	20	25	20	25	20	25

**Table 3:** Alloying parameters for samples of set II.

Sample	13	14	15	16	17	18	19	20
Pulses	10	10	20	20	30	30	50	50
EB (kV)	25	30	25	30	25	30	25	30

**Table 4:** Ra and Rq values for untreated and treated samples.

Sample	Ra (μm)	Rq (μm)
Etched	1.16 ± 0.07	1.18 ± 0.18
EB	0.48 ± 0.05	0.63 ± 0.11
5	0.30 ± 0.04	0.37 ± 0.05
6	0.33 ± 0.08	0.43 ± 0.11

**Table 5:** Crystallite size of set I samples.

Sample	Crystallite Size (nm)
Etched	92
EB	92
1	34
2	35
5	35
6	27
11	31
12	30

**Table 6:** Lattice parameters of orthorhombic α″ cell.

Sample	a (Å)	b (Å)	c (Å)	Cell Volume (Å^3^)
5	3.322	4.621	4.630	71.077
6	3.178	5.081	4.645	74.997
11	3.273	4.624	4.637	70.172
12	3.307	4.595	4.642	70.551

**Table 7:** Lattice parameters, cell volume and Nb content in *hcp* of Ti–Nb alloy.

Sample	a (Å)	c (Å)	Cell Volume (Å^3^)	Nb (at.%)
1	2.943	4.671	35.035	5.45
2	2.944	4.668	35.039	5.53
5	2.944	4.670	35.055	5.55
6	2.942	4.656	34.904	5.40
11	2.944	4.669	35.036	5.50
12	2.944	4.670	35.054	5.55
13	2.938	4.692	35.076	5.85
14	2.936	4.689	35.013	5.70
15	2.942	4.692	35.161	6.20
16	2.943	4.688	35.159	6.35
19	2.936	4.694	35.027	5.60
20	2.943	4.690	35.175	5.70

**Table 8:** Nb mean content calculated from GDOES data of set I.

Sample	Deposited Nb Thickness (nm)	Thickness of Alloy after LEHCEB (μm)	Nb Mean Content (at.%)
1	25	0.64	3.7
2	25	0.37	6.2
5	75	0.76	8.8
6	75	1.05	6.5
11	150	3.92	3.6
12	150	3.21	4.4

**Table 9:** Nb mean content calculated from GDOES data of set II.

Sample	Thickness of Alloy after LEHCEB (μm)	Nb Mean Content (at.%)
13	1.45	6.3
14	1.49	6.2
15	1.50	6.1
16	1.58	5.8
19	1.72	5.4
20	1.34	6.8

## Data Availability

Data sharing is not applicable for this article.
